# Development of the Patient’s Experience and Attitude Colposcopy Eindhoven Questionnaire (PEACE-q)

**DOI:** 10.1186/s12913-019-4425-2

**Published:** 2019-08-20

**Authors:** Victor J. M. Pop, Tirza Wouters, Ruud L. M. Bekkers, Viola R. M. Spek, Jurgen M. J. Piek

**Affiliations:** 1 Department of Medical and Clinical Psychology, Tilburg University, Tilburg, Netherlands; 2 Department of Obstetrics and Gynaecology, Catharina Hospital, Eindhoven, The Netherlands

**Keywords:** Colposcopy, Patient caregiver attitude, patient’s experience, Satisfaction, Anxiety

## Abstract

**Background:**

No validated instruments for the evaluation of patient satisfaction in colposcopy do exist. Therefore, this study reports on the development of a Patient’s Experience and Attitude to Colposcopy questionnaire.

**Methods:**

Patients who recently received colposcopy participated in a focus group. A panel of experts evaluated the transcriptions and agreed on a 15-item draft questionnaire. The draft questionnaire was completed by 68 women who subsequently came for a colposcopy. For construct validation, Exploratory Factor Analysis (EFA) and Confirmatory Factor Analysis (CFA) were performed as well as reliability analysis. Concurrent validity was assessed with the 4-item Patient Health questionnaire (PHQ-4).

**Results:**

Construct validation resulted in an 8-item patient perception scale with good psychometric properties (Cronbach’s alpha: 0.76) and excellent model fit. Two subscales could be discriminated: patient procedure perception scale (alpha: 0.89) and caregiver attitude perception scale (alpha: 0.71). Both subscales intercorrelated moderately (*r* = 0.28, *p* = 0.045). The subscale patient perception correlated significantly with the PHQ-4 scale and its anxiety subscale, not with the depression subscale.

**Conclusions:**

We developed a Patient’s Experience and Attitude to Colposcopy questionnaire with adequate psychometric properties. Future application in out-patient clinics should further evaluate its clinical relevance.

## Background

In the Netherlands, the prevalence of high risk Human Papilloma Virus (hrHPV) is 8% in women between 30 and 60 years [1]. These women are invited once every 5 years to participate in the national cervical screening program. In 2017, the program was changed to include primary hrHPV detection. As a result, the number of referrals to the gynaecologist increased from 7.741 in 2017 to an estimated 14.789 (+ 91%) in 2022 [2]. This increase in referrals has made it even more important to maintain patient’s satisfaction with colposcopy. Colposcopy is the standard procedure in diagnosing high-grade squamous intraepithelial lesions (HSIL), a precancerous stage of cervical cancer. This procedure is performed by a gynaecologist and is associated with dissatisfaction and anxiety in patients [3, 4]. Patient satisfaction plays a considerable role in the compliance, continuity of care and relationship with the caregiver [5, 6]. It is important to have a tool to investigate the level of satisfaction to improve and assure quality of care [7].

However, measuring patient satisfaction is complex due to multiple variables: the ambiguous definition of patient satisfaction, media influence and individual patient biases [8]. Different validated tools have been developed to explore the patient’s experience with given care. Examples of such tools are the Quality of Care Through the Patient’s Eyes (QUOTE) [9] and the Consumer Assessment of Healthcare Providers and Systems (CAHPS) [10] surveys. Unfortunately, these questionnaires are intended to measure on the meso-level of healthcare and are therefore not specific enough for assessing colposcopy experience [11]. While determinants of women’s psychological effects in colposcopy have been addressed before, no studies are available assessing the patient’s colposcopy satisfaction [12].

The objective of this study was to develop a user-friendly questionnaire to examine the patient’s colposcopy experience and investigate the psychometric properties: the Patient’s Experience and Attitude Colposcopy Eindhoven Questionnaire.

## Methods

### Procedure

Prior to developing a first draft scale, women who recently received colposcopy were invited to participate in a focus group interview. Assisted by a psychologist, staff members from the department of Medical and Clinical Psychology of Tilburg University conducted a 2 h lasting interview with 5 women. An open interview strategy was applied: the women were not offered predefined questions. Instead, they were invited to express their feelings and thoughts about the colposcopy examination. With permission of the participants, the interview was recorded. The recording was transcribed into texts, sorted by subject, and evaluated by a panel of experts (VS, JP and VP). According to this panel, there remained fifteen topics that were repeatedly mentioned by different women. These topics were transcribed into 15 questions and were reviewed on formulation and user-friendliness by the women who participated in the interview and by several care providers and resulted in a final draft for construct validation. These items especially referred to aspects of the way the procedure was perceived by the women: “I felt stressed before the colposcopy” and “I was worried about the outcome of the colposcopy”. Also, these items contained aspects how the woman perceived the attitude of the care-giver during the procedure: “The gynaecologist put me at ease” and “I was well-informed about the next steps after the colposcopy”. The questionnaire items were formulated in positive and negative statements and rated on a 4-point Likert scale (1 = totally agree and 4 = totally disagree). After recoding, higher scores indicated a more positive colposcopy experience.

### Participants

In 2017, a cohort of 74 women who underwent colposcopy at the department of Gynaecology and Obstetrics of the Catharina Hospital Eindhoven, the Netherlands were invited to complete the first draft questionnaire anonymously. Women were included if they received a colposcopy recently and were able to read and understand Dutch sufficiently. The ethical review board approved of this study. Prior to the study, all participants gave written informed consent.

### Measurements

The questionnaire consisted of 15 abovementioned items of the PEACE-q. In addition, several questions regarding demographics and gynaecological features were answered (age category, civil status, educational level and parity). Furthermore, the Patient Health Questionnaire-4 (PHQ-4) was completed. The PHQ-4 questionnaire was used to assess anxiety and depression and contained four key questions, rated on a 4-point Likert scale. This questionnaire was extensively validated for use in healthcare [13]. The PHQ-4 can be divided into two subscales: The Patient Health Questionnaire-2 (PHQ-2) anxiety subscale and the PHQ-2 depression subscale. Higher scores indicate more depressive or anxiety symptoms.

### Statistical methods

Statistical analyses were performed using the Statistical Package for Social Sciences (SPSS version 20, IBM, Chicago, IL, USA) for Exploratory Factor Analysis (EFA) and reliability analysis (Cronbach’s alpha). Parallel Analysis was performed using the MonteCarlo PA program [14]. The Confirmatory Factor Analysis (CFA) was done using AMOS (version 18, IBM, Chicago, IL, USA).

### Factor analysis

A principal component Exploratory Factor Analysis (EFA) with oblimin rotation was executed to test the psychometric properties of the 15-item draft questionnaire. To select factors for retention, a Scree Catell plot was utilised. A data matrix was randomly generated using criterion values corresponding with the Eigenvalues from the abovementioned EFA through a Parallel Analysis. Eigenvalues that exceeded the corresponding criterion values were processed exclusively [15]. Only factor loadings over 0.40 were considered relevant. When the difference of the factor loadings was at least 0.20, the items that did load on more than two factors were retained. Cronbach’s alpha was used for the total scale and possible subscales to execute the internal consistency analyses. The minimal acceptable criterion of instrument internal reliability is considered as Cronbach’s alpha of ≥0.70 [16]. A Confirmatory Factor Analysis (CFA) was conducted on the remaining items of the first draft scale. The CFA was used to test the model fit of the factor structures found with the EFA. The following components were assessed: the comparative fit index (CFI), the normed fit index (NFI), the Tucker-Lewis Index (TLI), and the root mean square error of approximation (RMSEA). An adequate model fit can be assumed with a CFI ≥ 0.80, combined with a NFI ≥ 0.80, a TLI ≥ 0.80, and a RMSEA ≤0.05 for good fit and ≤ 0.08 for adequate fit [17, 18].

### Concurrent and construct validity

The concurrent validity of the new scale was tested by correlating with the PHQ-4, using a two tailed Pearson’s correlation. The construct validity was tested using hypotheses according to comorbid correlation between depression or anxiety and PEACE-q scores using multivariate linear regression analysis, explained below.

## Results

### Exploratory factor analysis (EFA) and confirmatory factor analysis (CFA)

Of all invited women, 68 (92%) fully completed the questionnaire of whom the characteristics are shown in Table 1. The scores on all 15 items were normally distributed, as indicated by the skewness and kurtosis. All assumptions were met for conducting principal components analysis. The Kaiser-Meyer-Oklin value was > 0.60 (0.74) and the Bartlett’s test of sphericity value was significant (*p* <  0.001). The EFA with oblimin rotation and the Scree Catell plot revealed a two dimensions structure with Eigenvalues 4.2 and 2.9 respectively, with 47% total explained variance.

**Table 1:** Characteristics of the first draft sample (*n* = 68)

Demographics	n	%
Age		
20–30 years	6	9
31–40 years	17	25
41–50 years	22	32
51–60 years	13	19
61–70 years	10	15
Civil status		
With partner	37	54
Single	12	18
Divorced/split up	10	15
Long distance relationship	3	9
Widow	6	4
Educational level		
Primary education	13	19
Secondary education	25	37
Tertiary education	30	44
Parity		
Nulliparity	22	32
Primiparity	13	19
Multiparity (> 1)	33	49

Table 2 presents the Eigenvalues of the 15 items used in the first draft scale. Item 5 did not discriminate between the two dimensions mentioned above. Item 9 and item 12 did not load and were omitted. Another EFA of the remaining 12 items revealed a two dimensions structure with Eigenvalues 4.0 and 2.6 respectively, with 56% total variance explained. These 12 items were divided into two factors with 6 items each. Parallel analysis showed two components with Eigenvalues exceeding the corresponding criterion values for a randomly generated data matrix of the sample size (12 variables * 68). Reliability analysis (Cronbach’s alpha) of the 12-item scale showed an alpha of 0.77, which increased to 0.84 when item 11 and item 13 were omitted. As a result, 10 items were left representing two subscales: 6 items referred to the patient’s perception of the procedure (patient subscale) with alpha = 0.89 and 4 items referred to the patient’s perception of the communication by the caregiver and/or attitude of the caregiver (caregiver subscale) with alpha = 0.72. This 10-item draft was subsequently tested in a CFA and showed a poor model fit: CFI = 0.78, NFI = 0.78, TLI = 0.75 and RMSEA = 0.09.

**Table 2:** First draft of a colposcopy self-rating questionnaire (15 items)

	Eigenvalues of subscales	
Two-dimension structure	4.2	2.9
1. I was well-informed about the colposcopy		0.603
2. I was not looking forward to the colposcopy	0.718	
3. I was preoccupied with the possible outcome of the colposcopy	0.802	
4. I felt stressed about the colposcopy	0.817	
5. The colposcopy went as I expected	0.526	0.412
6. I was worried about the outcome of the colposcopy	0.840	
7. I was worried about the possible further treatment options	0.818	
8. The gynecologist put me at ease		0.629
9. I was pleased that I was able to watch the procedure along on the monitor		
10. I experienced the procedure as painful	0.673	
11. I am satisfied with the provided information about the colposcopy		0.775
12. I have done some research on the internet myself	0.316	
13. After completing the colposcopy, I was well-informed when it was permitted to have sexual intercourse again		0.606
14. I am satisfied about the way I received the results		0.742
15. I was well-informed about the next steps after the colposcopy		0.608

However, when item 2 and item 10 were omitted due to overlap in the item matrix, a final 8-item questionnaire remained with an excellent model fit in CFA: CFI = 0.92, NFI = 0.98 and RMSEA = 0.056 (lower bound of 0.01). One 4-item subscale referred to the patient’s perception of the procedure (alpha = 0.89) and the other 4-item subscale referred to the patient’s perception of the caregiver’s attitude (alpha = 0.72). Cronbach’s alpha of the total 8-item scale showed an alpha of 0.81. The final draft was tested with an EFA and it showed a two-factor structure with Eigenvalues of 3.4 and 1.8 (65% total explained variance), as shown in Table 3. The items were recoded from 1 to 4 into 0–3 in order to have an item range from 0 to 24. Higher scores indicate a more positive perception of the colposcopy procedure.

**Table 3:** Final 8-item PEACE scale

	Eigenvalues of subscales	
Two-dimension structure	3.4	1.8
1. I was well-informed about the colposcopy		0.664
2. I was preoccupied with the possible outcome of the colposcopy	0.895	
3. I felt stressed about the colposcopy	0.764	
4. I was worried about the outcome of the colposcopy	0.938	
5. I was worried about the possible further treatment options	0.843	
6. The gynecologist put me at ease		0.664
7. I am satisfied about the way I received the results		0.824
8. I was well-informed about the next steps after the colposcopy		0.769

### Concurrent validity and construct validity

Table 4 presents the correlations between the mean scores and range of the 8-item questionnaire (PEACE-8) and of the 4-item PHQ questionnaire, including correlations between the subscales. The total PEACE-8 correlated significantly and inversely with the PHQ-4 and the PHQ-2 anxiety scale. The PEACE-8 did not correlate with the PHQ-2 depression scale. The patient and caregiver subscales intercorrelated moderately although significantly (low effect size). The patient subscale and the PHQ-4 correlated moderately as well. In contrast, the caregiver subscale did not correlate with the PHQ-4 or its subscales.

**Table 4:** Correlations between the PEACE-8 (sub) scales, the PHQ-4 (sub) scale and mean (SD) scores

	Mean (SD)	Range	PEACE-8 total	PEACE-4 patient	PEACE-4 caregiver	PHQ-4 total	PHQ-2 anxiety	PHQ-2 depression
PEACE-8 total	9.3 (4.0)	0–17	–	0.91**	0.63**	−0.36**	−0.45**	− 0.15
PEACE-4 patient	6.8 (3.2)	0–12	0.91**	–	0.27*	0.43**	0.53**	−0.18
PEACE-4 caregiver	2.5 (1.7)	0–7	0.63**	0.27*	–	0.03	0.04	−0.03
PHQ-4 total	5.9 (2.3)	4–13	−0.36**	−0.43**	− 0.03	–	0.95**	0.86**
PHQ-2 anxiety	3.3 (1.5)	2–8	−0.45**	−0.53**	− 0.04	0.95**	–	0.64**
PHQ-2 depression	2.6 (0.9)	2–5	−0.15	−0.18	− 0.03	0.86**	0.64**	–

* p < 0.05; ** p < 0.01; SD Standard deviation*, PEACE-8 total* Patient’s experience attitude and colposcopy questionnaire*, PEACE-4 Patient* Patient perception subscale*, PEACE-4 care-giver* Patient perception of care-giver’s attitude subscale*, PHQ-4 total* Patient health questionnaire 4-item containing two main symptoms of depression and two symptoms of general anxiety*, PHQ-2 anxiety* Anxiety subscale of PHQ-4*, PHQ-2* Depression subscale of PHQ-4

Finally, we performed two multivariate regressions based on the significant correlations found at the univariate level. One of the multivariate regressions was calculated with the caregiver subscale as dependent variable and the PHQ-2 anxiety subscale as independent variable, adjusting for confounders (age, parity, educational level and civil status). Table 5 shows the results and indicates that higher age and higher education were independently related to higher scores on the caregiver subscale (F = 5.8, *p* <  0.001, total explained variance r^2^ = 0.27). The second multivariate regression was calculated with the patient subscale as dependent variable and the PHQ-2 anxiety subscale as independent variable, adjusting for abovementioned confounders. As seen in Table 6, anxiety scores were the highest negative predictor of high patient’s perception scores. Higher age and higher education were also significantly related to high positive perception scores (*F* = 6.4, *p* < 0.001, total explained variance *r*^*2*^ = 0.38).

**Table 5:** Multivariate linear regression, dependent variable: care-giver patient attitude scores

	B	Beta	T	*p*-value
Higher age	0.52	0.41	−3.50	0.001
Single	−0.89	− 0.20	1.77	0.08
High education	0.64	0.29	−2.53	0.01
Multiparity	0.07	0.04	−0.31	0.76
High anxiety	−0.03	−0.03	− 0.26	0.79

**Table 6:** Multivariate linear regression, dependent variable: patient’s perception score

	B	Beta	T	*p*-value
Higher age	0.575	0.250	2.058	0.045
Single	0.264	−0.34	0.290	0.773
High education	1.055	0.255	2.026	0.048
Multiparity	0.699	0.214	1.766	0.083
High anxiety	−0.960	−0.500	−4.511	< 0.001

## Discussion

This study is the first to develop and validate a questionnaire to examine the patient’s colposcopy experience following a rigorous protocol: focus group interview, first draft of questionnaire followed by construct validation including a Confirmatory Factor Analysis. The final draft of the PEACE-q contains 8 items, divided in two subscales, with adequate psychometric properties. The factor structure of both sub-scales met all the criteria of appropriate construct validation (appropriate Eigen values, explained variance and excellent model fit in a CFA). Moreover, internal consistency of the two subscales was high.

The two subscales of the PEACE-q were: the patient’s perception of the procedure and the patient’s perception of the caregiver’s attitude before and during the colposcopy. Since the items reflect different aspects of the women’s perception of the whole procedure, there was only a modest correlation between the two subscales. Furthermore, the patient perception subscale, containing items related to stress and worrying, correlated significantly with the PHQ-2 anxiety scale and not with the PHQ-2 depression scale. This explains why the PHQ-2 anxiety scale is the most important determinant of this PEACE-q subscale at a multivariate level. Moreover, for both PEACE-q subscales, it is important to emphasize the higher scores (more satisfaction) are related to higher age and higher educational level. Putting it in the opposite way, younger and lower educated women are at risk for poor scores.

The review by Kalucy et al. illustrates the difficulties in creating patient satisfaction surveys [8]. Our findings confirm the influence of individual factors such as education level and age on measuring patient satisfaction. The report by Robert et al. recommends to emphasize on quality improvement based on what we already know matters most to patients by developing solutions to meet patients’ needs [19]. However, this recommendation regards to general patient satisfaction surveys, not to specific patient satisfaction like in colposcopy.

The PEACE-questionnaire should be used in clinical practice to reveal the patients’ needs. Firstly, the current study suggests that young and low educated women are at risk for perceiving the colposcopy procedure as a most stressful experience. This should be considered when women are informed on the colposcopy procedure. Secondly, the current study shows that the attitude of the healthcare professional (gynaecologist, nurse, etc.) towards the patient is important and also the way she is informed on colposcopy.

A strength of the current study is the application of a rigorous protocol that should preferentially be used to develop new questionnaires: focus groups as the starting point to develop a draft questionnaire containing items that really are important for women. All items of the final PEACE-q came up spontaneously during the focus group interview and reflect aspects of the colposcopy examination which were regarded as important by the women (not the health professionals).

The present study had its limitations. Firstly, the scope of this study was limited in terms of the Dutch language. So the questionnaire should be translated to be used by women speaking different languages. Secondly, only five participants in the focus group limited the initial items and the sample size was relatively low. Future studies should repeat and further investigate the construct and concurrent validity of the PEACE-q.

It is well known that colposcopy after abnormal PAP smear needs a careful follow-up [20]. Also, it is well known that compliance to follow-up is depending on patient satisfaction concerning previous treatments [21, 22]. Therefore, the PEACE-q seems to be a clinical relevant instrument to detect patients at risk for poor compliance (as reflected by poor patient satisfaction). Also, the current study shows patients at risk for poor scores and who should receive special attention: young and low educated women. In clinical practice, special attention should be given to these sub-groups of women in terms of: education, explanation and a patient centred attitude of the caregiver during the procedure. Finally, the two different dimensions of the scale might help training healthcare professionals in order to improve patient satisfaction of the colposcopy procedure.

## Conclusions

The PEACE-q is a valid 8-item user-friendly self-rating tool with adequate psychometric properties. In addition, older and high educated women independently lead to higher scores on the caregiver subscale. Therefore, young and low educated women have to receive additional explanation and a more patient centred attitude.

## Data Availability

The datasets used and/or analysed during the current study are available from the corresponding author on reasonable request.

## Abbreviations

CAPHS: Consumer Assessment of Healthcare Providers and Systems
CFA: Confirmatory Factor Analysis
CFI: Comparative Fit Index
EFA: Exploratory Factor Analysis
hrHPV: high risk Human Papilloma Virus
HSIL: High-grade Squamous Intraepithelial lesions
NFI: Normed Fit Index
PEACE-8: The 8-item Patient’s Experience and Attitude Colposcopy Eindhoven
PEACE-q: Patient’s Experience and Attitude Colposcopy Eindhoven Questionnaire
PHQ-2: The 2-item Patient Health questionnaire
PHQ-4: The 4-item Patient Health questionnaire Questionnaire
QUOTE: Quality of Care Through the Patient’s Eyes
RMSEA: Root Mean Square Error of Approximation
SPSS: Statistical Package for Social Sciences
TLI: The Tucker-Lewis Index
